# CHALLENGES AND RECOMMENDATIONS TO MEETING THE PALLIATIVE CARE NEEDS OF RESIDENTS IN ASSISTED LIVING

**DOI:** 10.1093/geroni/igac059.1116

**Published:** 2022-12-20

**Authors:** Debra Dobbs, Hongdao Meng, William Haley, Carlyn Vogel, Harleah Buck

**Affiliations:** University of South Florida, Tampa, Florida, United States; University of South Florida, Tampa, Florida, United States; University of South Florida, Tampa, Florida, United States; University of South Florida, Tampa, Florida, United States; Csomay Center for Gerontological Excellence, Iowa City, Iowa, United States

## Abstract

Of the more than 800,000 assisted living (AL) residents in the U.S. approximately 25% remain until the end of life and can benefit from palliative care (PC) and hospice care. Although the AL population is the fasting-growing segment of hospice care, increasing by 40% in the past five years, ALs still have special challenges in providing quality end-of-life care. ALs have a lack of nurse staffing and adequate training about how to care for those with serious illnesses. We present findings from studies over the last decade in Florida ALs (N=100) related to PC and hospice care practices. Some of the findings reveal gaps in care related to PC staff education, documentation of pain and advance directives and recognizing the need for more family involvement in decisions, especially for those with dementia. Recommendations on how to improve processes of care for residents who can benefit from PC will be discussed.

